# An Investigation of Social Ecological Barriers to and Facilitators of WIC Farmers Market Nutrition Program Voucher Redemption

**DOI:** 10.3390/nu14091871

**Published:** 2022-04-29

**Authors:** Renata Blumberg, Emily Fowler, Yeon Bai, Pankaj Lal, Alyssa Smolen, Ilana Dubrovsky

**Affiliations:** 1 Department of Nutrition and Food Studies, Montclair State University, Montclair, NJ 07043, USA; emilyfowler27@gmail.com (E.F.); baiy@montclair.edu (Y.B.); smolena1@montclair.edu (A.S.); ilana.dubrovsky@gmail.com (I.D.); 2 Department of Earth and Environmental Studies, Clean Energy and Sustainability Analytics Center, Montclair State University, Montclair, NJ 07043, USA; lalp@montclair.edu

**Keywords:** social ecological model, Farmers Market Nutrition Program, food access, farmers’ markets, local food

## Abstract

In the United States, many communities lack sufficient access to fresh produce. To improve access to fresh fruits and vegetables, the Special Supplemental Nutrition Program for Women, Infants, and Children (WIC) provides eligible participants vouchers through the Farmers Market Nutrition Program (FMNP) that can be redeemed directly from farmers at markets or farm stands. However, FMNP voucher redemption rates in New Jersey remain lower than those in neighboring states. This article used the social ecological model to examine differences between FMNP participants who redeem vouchers (Redeemers) and those who do not (non-Redeemers) in the areas of: produce procurement practices and consumption frequency, and barriers to and facilitators of FMNP voucher redemption. This cross-sectional study included WIC FMNP participants (*N* = 329) in northern New Jersey, USA. Analyses were conducted using descriptive statistics, independent sample *t*-tests, and one-way ANOVA. Compared to Redeemers, non-Redeemers consumed fewer average daily vegetable servings, were more likely to shop at small grocery/corner stores, and encountered significant barriers to FMNP redemption, e.g., difficulty finding time to redeem vouchers.

## 1. Introduction

In the United States, the prevalence of diet-related chronic diseases continues to be a problem, with higher rates found among Hispanic, African American, and low-income populations [1,2]. A troubling tendency has been the increasing incidence of Type 1 and Type 2 diabetes in youths [3]. While chronic diseases are associated with diverse causal factors, high fruit and vegetable consumption helps to reduce disease risk [4]. However, many communities across the United States lack sufficient access to fresh produce [5].

Over the years, United States federal food and nutrition programs have been modified to encourage fruit and vegetable consumption, and non-profit organizations have also initiated programs to improve access to fresh produce [6,7,8]. WIC is the third largest federal food and nutrition program in the United States, serving approximately 6.2 million individuals each month [9]. The program supports low-income women, infants, and children up to five years of age who are deemed nutritionally at-risk by a health care professional. Research demonstrates that the WIC population is at a greater risk of developing adverse health outcomes due to insufficient nutrition and inadequate dietary patterns, leading to micronutrient deficiencies among other nutrition-related health concerns [10,11].

WIC provides participants with group and individualized nutrition education, supplemental foods to increase consumption of fresh fruits and vegetables, and health care and social services referrals [12]. WIC participants also receive cash-value vouchers to purchase eligible foods from participating grocery stores and supermarkets; items include breakfast and infant cereals, fresh fruits and vegetables, whole wheat bread and other whole grains, eggs, and infant formula [13]. WIC’s objective is to provide nutrition and health care interventions early in life to improve long-term health outcomes.

The WIC Farmers Market Nutrition Program (FMNP) was founded in 1992 with the mission to provide fresh produce to WIC participants and expand access to farmers’ markets [14]. In 2015, WIC FMNP supported 1.7 million families with fresh produce; the program also provided over USD 14 million to over 18,000 participating farmers [15]. To redeem produce at farmers’ markets or farm stands, participants use FMNP vouchers to purchase fresh fruits and vegetables. FMNP vouchers may be redeemed from May until November, which marks the end of the season, while other WIC vouchers may be redeemed throughout the year [16].

State agencies have the authority to determine the value of FMNP vouchers. Federal FMNP guidelines enforce that the minimum benefit level must not fall below USD 10 and may not exceed USD 30, but states may choose to provide benefits either per individual or per family unit [17,18]. For example, New Jersey WIC FMNP vouchers were historically distributed once per year in the form of two USD 10 vouchers for each eligible participant (including the year when this research was conducted), but the value recently increased to USD 25 per participant for the season [19,20]. Other states such as South Carolina provide WIC FMNP participants with five USD 5 vouchers (USD 25 total) to purchase produce at approved farmers’ markets and farm stands [21].

The FMNP enables participants to select their own fresh, unprepared fruits, vegetables, and/or herbs from participating certified farmers. Although the number of locations to redeem vouchers (e.g., farmers’ markets and other direct-to-consumer marketing channels) has increased in New Jersey, redemption rates for WIC FMNP have remained below average in the region. In 2014, the FMNP redemption rate in New Jersey was 47.9%, while rates in surrounding states such as Pennsylvania and New York were 59.1% and 56.0%, respectively [22].

General FMNP redemption barriers mirror the barriers that low-income consumers face when attempting to shop at farmers’ markets [23,24]. However, few research studies have specifically focused on farmers’ market patronage by WIC participants, or by low-income consumers and ethnic/racial minorities more broadly [25]. In a systematic review of 49 peer-reviewed articles on facilitators of and barriers to farmers’ market patronage, Freedman et al. found that only 15% of the articles focused on racial or ethnic minorities, and 39% included information on low-income consumers [25]. Because farmers’ markets are relatively inexpensive to establish and have low overhead costs, compared to retail establishments, they provide an ideal structure to serve the nutritional needs of low-income communities with low access to fresh fruits and vegetables. However, considerable barriers hamper farmers’ market patronage among low-income consumers, including accessibility; transportation constraints; perception of high prices; lack of knowledge about WIC FMNP redemption possibilities; and inconvenient opening hours [25,26].

Because barriers to and facilitators of FMNP voucher redemption include individual, organizational, community, and policy-based factors, the social ecological model (SEM) provides a useful framework with which to understand FMNP redemption dynamics. The SEM conceives of individuals as shaped by social and spatial systems, which are conceptualized as concentric levels of influence around any given individual. Evaluating redemption behaviors through the lens of the SEM enables researchers to analyze the range of factors and themes that influence fruit and vegetable consumption. Applications of the SEM in nutrition research examine the significance of the following levels: first, the individual level of knowledge, habits, and beliefs; second, the interpersonal level of social relations, such as relationships between families and groups; third, the organizational level, including formal and informal organizations; fourth, community influences; and fifth, social structures and systems, including policies at all scales [27,28,29]. This framework has been widely used in nutrition research to better understand multiple determinants of behaviors through various levels of influence [30,31]. Analyzing behaviors within an individual’s social and spatial contexts enables researchers to identify influences at each level and to determine appropriate interventions [32].

Both barriers to and facilitators of FMNP redemption may exist at any of these levels. Individuals may already have the habit of going to the farmers’ market, which would facilitate FMNP redemption. Conversely, individuals may also lack knowledge on how to cook the fresh produce that is available at the farmers’ market, presenting a critical barrier. At the interpersonal level, family members may influence FMNP redemption due to their taste preferences. At the organizational level, the farmers’ market may not have convenient opening hours. At the community level, the farmers’ market may be located too far away. Finally, at the policy level, the WIC FMNP is a federal program, but states administer the program and determine eligibility requirements for farmers as well as the value of the FMNP vouchers. Therefore, state-level policies may influence redemption. For example, at the time of this study, New Jersey farmers were required to have at least five acres in production to certify becoming WIC FMNP farmers [19,33]. In addition, other state and local policies may also be influencing redemption. For example, states or local municipalities may play an active role in supporting the creation of farmers’ markets, thereby improving redemption possibilities in their communities.

Despite the existence of these documented barriers, researchers have found that farmers’ market patronage is associated with higher fruit and vegetable consumption [34,35], and participation in the FMNP program has a positive effect on vegetable consumption [36]. However, sufficient research has not been conducted to understand why many WIC participants do not redeem their FMNP vouchers.

The goal of this research project was to analyze FMNP voucher redemption by WIC participants, a population that is nutritionally at-risk and low-income. In particular, we sought to identify differences between FMNP voucher recipients who redeemed their vouchers and those who received FMNP vouchers but did not redeem them. Specifically, we sought to examine differences in demographic characteristics, produce procurement and consumption practices, and self-reported FMNP redemption barriers and facilitators. We hypothesized that, in comparison to WIC participants who did not redeem their FMNP vouchers, WIC participants who redeemed their FMNP vouchers would have similar demographic and behavioral characteristics to farmers’ markets shoppers more broadly. We also hypothesized that WIC participants who did not redeem their FMNP vouchers would report more barriers to FMNP redemption at all levels of the SEM. The research presented here is part of a larger research project. The findings from the larger project have been presented in poster sessions at US conferences and are published as abstracts [37,38].

## 2. Materials and Methods

A cross-sectional survey for WIC participants in northern New Jersey was conducted between October 2017 and January 2018. These months were selected because they coincide with the end of the farmers’ market season. This study design enabled the researchers to investigate multiple variables (e.g., redemption barriers and facilitators) at a single point in time. A non-randomized, convenience sampling method was utilized to increase the number of responses, and because randomization would have been difficult given that we did not have contact information for all FMNP participants. The survey was conducted utilizing an electronic tablet using the Qualtrics Survey Software offline tablet application, and the survey took approximately 10 to 15 minutes to complete. To recruit participants, a plea was given to individuals in two WIC office waiting rooms during opening hours. Both the plea and survey were written in the English and Spanish languages in consideration of local demographics; Spanish is the second most prevalent language spoken in households among all New Jersey counties and provided the opportunity for greater participation in this study [39]. Furthermore, the New Jersey Department of Health’s Division of Family Health Services estimates that over 50% of the WIC population in the state are Latino/Hispanic, and the primary language spoken in 30% of WIC households is Spanish [40]. Incentives were offered to participants, including a snack, stickers for children, and an opportunity to enter a gift card draw for a local supermarket. Institutional review board approval was granted prior to beginning data collection.

The survey was divided into three parts. The first section assessed fruit and vegetable procurement and consumption practices and values, such as where and how often individuals shop for fresh fruits and vegetables between the months of June and November, the time period when FMNP vouchers are valid. Another question covered the transportation modes typically used to travel to the store or market. The importance of store or market characteristics, such as opening hours, location, accessibility, and measures of convenience, were assessed on a 5-point Likert scale (1 = Very Unimportant to 5 = Very Important). Fruit and vegetable consumption was self-reported on two scales. An adapted 2-item serving screener for fruit and vegetable intake was used [41]. Sample serving sizes were described and photos were provided to illustrate different serving sizes. Average fruit and vegetable consumption was assessed on a scale of “None” or “Less than One” (calculated as 0 average daily servings), “One” (calculated as 1 average serving), “Two” (calculated as 2 average daily servings), etc. Second, the number of servings of fruits and vegetables consumed in the past 24 hours was assessed, allowing respondents to specify a specific integer (0, 1, 2, 3, etc.). Additionally, a self-reported assessment of diet quality was obtained, including rankings of Poor, Fair, Good, Very Good, and Excellent. Common barriers to fruit and vegetable procurement and consumption were presented to respondents, such as price, time to prepare, and likeability of vegetables. Subsequent sections included questions on FMNP participation in previous years. Finally, the survey assessed demographic characteristics such as age, gender, education level, race/ethnicity, and employment status. The survey tool was developed and pre-tested to elicit feedback and to estimate response rates.

Upon the completion of data collection, the results were exported into IBM Statistical Package for Social Sciences (SPSS) Version 25 for analysis. Survey respondents who participated in the FMNP in the previous year (2017) were clustered into two groups based on redemption behavior: those who redeemed any or all of their vouchers (Redeemers), and those who did not redeem any vouchers at all (non-Redeemers). One-way ANOVA and independent sample *t*-tests were conducted to compare mean redemption rates between Redeemers and non-Redeemers, grouped by demographics, barriers, and facilitators. Open-ended comments submitted by non-Redeemers were coded for themes [42], such as the type of barrier (e.g., time, interest, or knowledge) that respondents experienced in redeeming FMNP vouchers. Some respondents listed multiple barriers. Each barrier was counted in its specific category. Qualitative coding was cross-checked by another member of the research team. In addition, the FMNP redemption rate was calculated via the self-reported total value of vouchers redeemed divided by the total value of vouchers received for each participant. For example, if an individual received two USD 10 vouchers and only redeemed one of them, that individual’s redemption rate would be calculated as 50.0%. The redemption rates were also calculated for different demographic groups and analyzed using one-way ANOVA.

## 3. Results

A total of 333 respondents indicated that they received FMNP vouchers in 2017. Of the FMNP participants, 228 redeemed all or some of their vouchers (henceforth “Redeemers”) and 101 did not redeem any of their vouchers (henceforth, “non-Redeemers”). Four respondents did not indicate whether they redeemed or not and were therefore excluded from the analysis. A total of 152 (46.2%) respondents took the survey in English and 181 (55.0%) took the survey in Spanish.

The overall FMNP redemption rate in the 2017 season was 63.2%. Survey respondents who were over the age of 35 had a higher FMNP redemption rate, with a mean redemption rate of 72.3% (see Table 1). FMNP redemption rates were lower for survey respondents aged 25 to 34 and the lowest for those under the age of 24. The differences between these age groups were statistically significant (*p* = 0.041). While not significantly different (*p* = 0.251), FMNP recipients with higher levels of education had lower redemption rates (see Table 1). FMNP recipients employed on a part-time basis had a higher redemption rate than those who were employed full-time or unemployed, although this difference was also not statistically significant (*p* = 0.550).

Respondents were asked to report their fruit and vegetable consumption as a daily average. In addition, total servings of fruits and vegetables consumed in the last 24 hours were recorded. As a daily average, Redeemers indicated eating more servings of vegetables than non-Redeemers, at a statistically significant level (1.66 versus 1.43, *p* = 0.050) (see Table 2). Redeemers reported slightly higher daily average consumption of fruit, but this difference was not statistically significant (1.94 versus 1.87, *p* = 0.520).

Redeemers were significantly more likely to agree that both fruits and vegetables are too expensive. Although non-Redeemers reported a higher level of agreement with the statement that vegetables are time-consuming to prepare, this difference was not significant. Individuals were also asked where they usually shop for fresh fruits and vegetables between the months of June and November, and they could check as many or as few retail outlets from a list of 12 options. Fewer Redeemers than non-Redeemers shopped at supermarkets for fruits (64.0% versus 70.3%) or superstores (18.9% versus 26.7%). However, a greater proportion of non-Redeemers shopped at small grocery/corner stores (30.7% versus 23.2%).

In comparison to Redeemers, non-Redeemers frequented fewer types of retail outlets to purchase fresh fruits and vegetables (2.09 versus 2.46 retail outlets). Similarly, Redeemers were more likely to utilize a greater number of transportation methods to travel to the store/market, but this difference was not significant. For Redeemers, these included private cars (57.5%), walking (39.0%), public bus (19.3%), and taxi/rideshare (16.2%). In contrast, non-Redeemers relied on private cars (65.3%), walking (35.6%), taxi/rideshare (11.9%), and public bus (9.9%).

Table 3 displays FMNP redemption barriers, which were averaged and compared between groups. Non-Redeemers encountered barriers to FMNP redemption at the individual level, which were significantly higher than the individual-level barriers encountered by Redeemers. Table 4 displays FMNP redemption facilitators. Redeemers were more likely to agree that they would redeem the vouchers if the value given per person was higher. In contrast, the average response reported for non-Redeemers was closer to neutral. Participants’ comfort level in redeeming the FMNP vouchers is analyzed in Table 3 and Table 4 at the interpersonal level and was found to be a statistically significant barrier to voucher redemption (*p* = 0.003). Participants also reported that they would redeem the vouchers if they were more comfortable in redeeming them. This was also found to be statistically significant (*p* = 0.016). Table 5 contains coded, open-ended comments provided by non-Redeemers, which provide insight into why they did not redeem their vouchers. High percentages of non-Redeemers encountered individual-level barriers.

## 4. Discussion

At the individual level of the SEM, important differences in demographic characteristics, knowledge, and practices were found between FMNP Redeemers and non-Redeemers. Older respondents had higher FMNP redemption rates, which confirms findings from other studies that found that farmers’ markets attract middle-aged adults [43]. Existing research has also shown that race/ethnicity may influence farmers’ market patronage, and that women and college-educated shoppers are more likely to shop at farmers’ markets [43,44,45,46,47,48]. In contrast, our study found that FMNP redemption rates decreased in groups with higher levels of education, although these differences were not statistically significant (Table 1). Interestingly, redemption rates were higher for those who worked part-time, in comparison to those who were unemployed or worked full-time, although this difference was not statistically significant. The inability to make time in personal schedules to go to the farmers’ market or farm stand was mentioned by 23.0% of non-Redeemers as a reason for why they did not redeem the vouchers (Table 5).

Another set of differences between Redeemers and non-Redeemers at the individual level includes fruit and vegetable consumption and procurement practices. Existing research on farmers’ market consumers has shown that they tend to consume more fruits and vegetables than shoppers who do not frequent farmers’ markets [35]. Other research on FMNP showed that after an intervention, individuals who redeemed the vouchers consumed more fruits and vegetables [44]. Our study found that, on average, Redeemers consumed more vegetables, but not significantly more fruit. Non-Redeemers were less likely to agree that fruits and vegetables were too expensive. Therefore, they may have fewer financial incentives to redeem the vouchers. Non-Redeemers were also more likely to report that it was difficult to find time to redeem vouchers (Table 3), and they were more likely to agree that they would redeem the vouchers if they had more time (Table 4). Other researchers studying dietary practices of low-income families have found that the perceived lack of time is a barrier to healthy eating [49,50]. 

Shopping strategies and transportation methods may also impact FMNP voucher redemption because low-income consumers face particular barriers when shopping for food [51]. For example, they are less likely to have access to a car than the general public [52]. Transportation has been an identified barrier in FMNP redemption as early as 1999, when the Community Food Security Coalition investigated whether transportation serves as a significant barrier to farmers’ market access [53]. In comparison to low-income American residents more broadly [51], our survey’s non-Redeemer respondents reported using a private car less often but walked more often. Faced with various barriers, low-income consumers develop shopping strategies which are highly dependent on access to different modes of transportation, including taxis [51]. Limited access to public transportation has been reported as a significant barrier for low-income consumers to accessing farmers’ markets [25,53]. In our study, most survey respondents selected more than one transportation method when asked how they usually travel to the store/market to purchase fresh fruits and vegetables. However, a minority of respondents reported using public buses, the main form of public transportation available in the surveyed communities. In comparison to Redeemers, among non-Redeemers, a higher percentage of respondents reported using a private car and a lower percentage of respondents reported walking or using a public bus. For 10.0% of non-Redeemers, transportation issues were a barrier to voucher redemption, and 8.9% explained that factors at the interpersonal level, such as having to take care of small children, influenced their transportation and shopping behavior (Table 5). Additional research demonstrates that a consumer’s proximity to farmers’ markets may influence where they shop; proximity may increase access and decrease barriers to fruit and vegetable consumption [34,54]. Furthermore, non-Redeemer responses indicated that they live further from farmers’ markets, increasing their need for alternate modes of transportation.

At the interpersonal level, we found that a lack of comfort in redeeming vouchers may play a role in voucher redemption. Martin et al. analyzed redemption rates among food stamp recipients as well as those eligible to visit a food pantry or soup kitchen [55]. This study discovered that one of the primary reasons this population does not visit a pantry or soup kitchen was due to the low comfort level participating in the program. Researchers state that addressing social stigma and acceptability could help to address these barriers to program participation.

At the organizational level, previous research has documented factors related to farmers’ market operations, which inhibit farmers’ market patronage by low-income consumers [25]. For example, low-income consumers may not frequent farmers’ markets because they do not offer a sufficient variety of foods or culturally appropriate food, or do not accept food stamps [25,56]. In a recent study analyzing FMNP participant barriers and behaviors in North Carolina, 76% of participants reported that variety was a primary motivator to redeeming FMNP vouchers [15]. Purchasing behaviors were also analyzed in this group and demonstrated that participants purchased over 20 different varieties of fruits and vegetables, with the most popular items being tomatoes, squash, bell peppers, and white potatoes. In our study, both Redeemers and non-Redeemers tended to disagree or have neutral opinions about statements such as: “the closest farmers’ market/farm stand lacks familiar produce” (Table 3). Our research found that although barriers at the organizational level did exist, more barriers at the individual level were found to be statistically significant. However, they may also be interrelated. For example, non-Redeemers reported significantly higher levels of agreement with the statements: “It is difficult to find time to redeem the vouchers,” and “The closest farmers’ market/farm stand is not open at convenient times.” Research conducted by Ball and colleagues also demonstrated that convenience in terms of opening hours and location is a considerable barrier for WIC FMNP participants, and reducing these barriers is critical to improving fruit and vegetable consumption in this vulnerable population [15]. Another recent study surveyed WIC FMNP participants and found that 35.65% of participants lacked knowledge of when the farmers’ market was open and 45.21% did not know of a farmers’ market in their local area [57]. Improved communication and consistency of farmers’ market opening hours and locations, as well as an increase in mobile farm usage, could be beneficial in reducing transportation and other barriers that participants face.

At the community level, one WIC office was served with a weekly mobile vegetable market operated by a local non-profit organization, and the other WIC office was in a city with a weekly farmers’ market. Nevertheless, non-Redeemers expressed a high level of agreement with the statement that “the closest farmers’ market/farm stand is too far away” (Table 3). This may be because WIC participants do not necessarily reside in the same city where the WIC office and farmers’ market are located. An Oregon case study analyzed 108 participant responses and found that 21% claimed that farmers’ market distance and limited opening hours were barriers to FMNP redemption [58]. Other research has shown that projects that make farmers’ markets more accessible, such as farm-to-WIC interventions, can improve voucher redemption and can be cost effective [59].

At the policy level, non-Redeemers reported lower levels of agreement with the statement that the value of the vouchers is not high enough. Specifically, the mean response was 2.85, which was between disagree and neutral. This suggests that increasing the value of the vouchers might not be enough to encourage non-Redeemers to utilize them. This has ramifications for programs such as the new incentive initiatives that provide extra vouchers to WIC and Supplemental Nutrition Assistance Program (SNAP) recipients who shop at farmers’ markets. These programs are meant to encourage low-income consumers to shop at farmers’ markets and therefore increase access to local food. While the value of the incentive varies, often these programs will double the value of what the shopper spends at the market using their WIC Cash Value Vouchers, FMNP vouchers, or SNAP benefits. Double-value programs provide benefits to farmers and consumers who use them [60], and research suggests that these programs may attract new farmers’ market patrons [61]. 

### Strengths and Limitations of This Study

Our research found significant individual-, organizational-, and community-level barriers for non-Redeemers. Some barriers at the individual level can be addressed more easily than others. For example, because non-Redeemers were more likely to agree that it is difficult to know where to redeem the vouchers, interventions designed to address this could include publishing the list of farmers who are certified to accept FMNP vouchers in a format that is easy to navigate. The state of New Jersey has only recently begun to publish this information, but other states provide this information on easily accessible websites. Information could be provided in multiple languages, with links to websites for the farms and farmers’ markets. Although some public listings in New Jersey include information on farmers’ markets that have farmers who accept FMNP, providing information on which farmers at the market accept FMNP would make the process of redeeming vouchers easier for WIC FMNP participants. Another individual-level barrier was the lack of time, an issue faced by families of diverse socioeconomic backgrounds [62]. A related organizational-level barrier was the inconvenient opening hours of farmers’ markets or farm stands. Weekend markets may be more attractive for this population, but these markets may experience problems attracting farmers, especially if they intend to be open for long hours. Participants were less likely to report a lack of quality, variety, or familiar produce as a barrier. In addition, in the open-ended responses, language barriers were not widely reported to be a barrier. This should be investigated further.

This study has several limitations. This cross-sectional study was designed to gain a large number of survey responses from WIC participants in urban areas of northern New Jersey. The goal was to provide a snapshot of redemption behaviors, rather than track participants over a period of time. In addition, surveys were conducted at only two WIC offices, and all data were self-reported. This type of study design may introduce bias. For example, it was not feasible to verify if the vouchers were, in fact, redeemed by the survey respondents. This study utilized non-randomized, convenience sampling, which may also introduce bias and limits the generalizability of the sample population. Future research could utilize other methods to track fruit and vegetable consumption and voucher redemption so that differences between Redeemers and non-Redeemers could be more accurately analyzed. Moreover, the generalizability of the findings is limited because each community will have different access to farm stands or farmers’ markets where vouchers could be redeemed. Future research could evaluate differences within the Redeemers category, such as by analyzing redemption rates among low and high Redeemers, or those that redeemed some or all of their vouchers. Barriers and facilitators can also be further analyzed to see if there are differences between groups based on their level of agreement with barriers to and facilitators of FMNP redemption. Farmer perspectives, another component of the larger issues regarding FMNP redemption, deserve further study.

Finally, this study was conducted before the coronavirus pandemic, which has had a significant impact on food insecurity [63], food procurement practices [64], and local food markets [65]. These impacts and resulting changes should be considered in any future research. Despite these changes and challenges, WIC and FMNP continued operating. Farmers’ markets adapted to meet coronavirus safety procedures, such as changing operations and infrastructure to practice social distancing. However, redemption barriers still exist. Certain barriers such as cost, availability of produce, and access to fruits and vegetables may have worsened during the pandemic, as global and domestic supply chains were significantly disrupted [66]. Future research could investigate any changes to redemption barriers since the start of the coronavirus pandemic. A variety of interventions are currently being implemented to increase FMNP redemption. Mobile markets are also becoming more popular with urban agriculture organizations who want to better serve their communities. Further research could explore differences between different interventions. For our study, we did not collect the addresses of the survey respondents. As a result, we were unable to more accurately link FMNP redemption with accessibility to a farmers’ market or farm stand. Future research could also take a place-based approach to redemption barriers so that interventions to improve redemption rates could be tailored towards individual communities.

## 5. Conclusions

According to the Centers for Disease Control and Prevention, only about 1 in 10 US adults consume the minimum amount of recommended daily fruits and vegetables [67]. The USDA WIC Farmers Market Nutrition Program was created to increase access to local produce for WIC participants [14]. The increasing use of electronic benefit transfer systems at farmers’ markets and the use of incentives have improved patronage among low-income consumers, including WIC participants, but a variety of barriers to redemption still remain [54,68,69]. Utilizing the social ecological model, this study identified FMNP redemption barriers, facilitators, and related behaviors in a population of WIC participants in the US state of New Jersey.

## Figures and Tables

**Table 1:** Demographics and Redemption Rates for Survey Respondents.

	*n*	Mean Redemption Rate (%)	P-Value
**Age**			
Under 24 years	62	54.44	0.041
25–34 years	155	62.15	
Over 35 years	100	72.25	
**Race/Ethnicity**			
Asian/Pacific Islander	5	90.00	0.193
Black or African American	50	62.00	
Hispanic or Latino	239	64.44	
Native American	1	0.00	
White	13	44.87	
Other	6	66.67	
**Highest Level of Education**			
Less than High School	43	71.51	0.251
High School Graduate, Diploma, or Equivalent (GED)	147	66.67	
Post High School Education, No Degree	88	59.75	
College Degree or More	35	54.29	
**Employment Status**			
Employed Full-Time	70	63.21	0.550
Employed Part-Time	40	71.25	
Unemployed	208	62.78	
One-way ANOVA tests were conducted to identify if there were any statistically significant differences in redemption rates between demographic groups.			

**Table 2:** Fruit and Vegetable Consumption.

	*n*	Mean	SD	P-Value
Average Daily Fruit Servings *				
Redeemers	228	1.94	0.97	0.520
Non-Redeemers	101	1.87	1.05	
Average Daily Vegetable Servings *				
Redeemers	227	1.66	0.96	0.050
Non-Redeemers	101	1.43	0.99	
24-Hour Recall: Fruit Servings *				
Redeemers	228	2.17	1.66	0.989
Non-Redeemers	101	2.17	1.66	
24-Hour Recall: Vegetable Servings *				
Redeemers	228	1.80	1.50	0.893
Non-Redeemers	101	1.77	1.86	
Independent samples *t*-tests were conducted to determine if there were significant differences in fruit and vegetable consumption between Redeemers and non-Redeemers. * 0 = Zero Servings or Less than One Serving; 1 = One Serving; 2 = Two Servings; 3 = Three Servings.				

**Table 3:** Barriers to FMNP Redemption.

Barriers to FMNP Redemption	Redeemers	Non-Redeemers	P-Value
	*n* = 228	*n* = 101	
	Mean (SD)	Mean (SD)	
**Individual**			
It is difficult to...			
...know where to redeem the vouchers	2.85 (1.49)	3.51 (1.50)	<0.001
...find time to redeem the vouchers	2.85 (1.45)	3.60 (1.40)	<0.001
**Interpersonal**			
It is uncomfortable to redeem vouchers	2.37 (1.36)	2.87 (1.50)	0.003
**Organizational**			
The closest farmers’ market/farm stand...			
...is not open at convenient times	3.56 (1.40)	3.98 (1.12)	0.010
...lacks quality produce	2.56 (1.34)	2.87 (1.22)	0.077
...lacks a variety of produce	3.10 (1.45)	3.12 (1.30)	0.920
...lacks familiar produce	2.94 (1.43)	2.94 (1.28)	0.969
...lacks produce that I or my family likes	2.90 (1.44)	2.90 (1.26)	0.979
**Community**			
The closest farmers’ market/farm stand is too far away	3.26 (1.47)	3.78 (1.28)	0.003
**Policy**			
The value of the vouchers given per person is not high enough	3.07 (1.43)	2.85 (1.36)	0.196
1 = Highly Disagree, 2 = Disagree, 3 = Neutral, 4 = Agree, 5 = Highly Agree. Independent samples *t*-tests were conducted to determine if there were significant differences in mean barriers to FMNP redemption between Redeemers and non-Redeemers.			

**Table 4:** Facilitators of FMNP Redemption.

“I would redeem if...”	Redeemers	Non-Redeemers	P-Value
	*n* = 228	*n* = 101	
	Mean (SD)	Mean (SD)	
**Individual**			
I knew where to redeem the vouchers	3.46 (1.45)	4.00 (1.24)	0.002
I had more time to redeem the vouchers	3.59 (1.40)	4.04 (1.14)	0.006
**Interpersonal**			
I was more comfortable redeeming vouchers	3.35 (1.39)	3.75 (1.20)	0.016
**Organizational**			
the closest farmers’ market/farm stand...			
...was open at more convenient times	3.88 (1.28)	4.25 (0.88)	0.012
...had better quality produce	3.34 (1.46)	3.44 (1.23)	0.624
...had a better variety of produce	3.71 (1.36)	3.54 (1.24)	0.332
...carried more familiar produce	3.67 (1.38)	3.59 (1.19)	0.680
...had produce that I or my family likes	3.64 (1.38)	3.61 (1.19)	0.872
**Community**			
the closest farmers’ market/farm stand was located closer	3.94 (1.22)	4.14 (0.97)	0.168
**Policy**			
the value of the vouchers given per person was higher	3.82 (1.31)	3.32 (1.38)	0.002
1 = Highly Disagree, 2 = Disagree, 3 = Neutral, 4 = Agree, 5 = Highly Agree. Independent samples *t*-tests were conducted to determine if there were significant differences in mean facilitators to FMNP redemption between Redeemers and non-Redeemers.			

**Table 5:** Analysis of Open-Ended Responses.

Barriers to FMNP Redemption		% Non-Redeemers	Examples
		Reporting Barrier	
	*n*	*n* = 90	
**Individual**			
Lack of...			
...time	23	25.6%	“No time to make a special trip.”
			“I work 7 am to 4 pm. It is impossible for me to redeem my checks if the veggie car [mobile farm stand] only comes Friday mornings.”
...interest	8	8.9%	“I forgot I had them.”
...knowledge on how to redeem/difficult finding where to redeem	33	36.7%	“Figuring out where to go, commuting may be difficult.”
			“We couldn’t find a farmers market in our area.”
...access to private car/transportation issues	9	10.0%	“No ride”
Other personal barrier	7	7.8%	“Line too long, bags too heavy, pregnant.”
**Interpersonal**			
Children limit shopping opportunities	8	8.9%	“I don’t have a car; it’s not local enough for me having a 3 year old and her things to take on a bus.”
Language barrier limits shopping opportunities	1	1.1%	“Because I wasn’t sure of the expiration date, language barrier.”
**Organizational**			
Farmers’ market/farm stand...			
...is not open at convenient times	11	12.2%	“Timing of the markets. Extend opening hours.”
...is not reliable (hours; produce gets sold out)	8	8.9%	“[The mobile farm stand] is not reliable; at times they don’t show up.”
...lacks variety or familiar food	5	5.6%	“They don’t bring enough products I’m familiar with.”
**Community**			
Community lacks...			
...farmers’ market/closest market is too far	27	30.0%	“I have no time; the nearest market it’s far; I usually walk there.”
...adequate transportation options	8	8.9%	“The places were too far; I didn’t know how to get there. Taxi charges me more than $20, more than the value of the checks.”

## Data Availability

Not applicable.
